# Supplementary material for: Chiral multiferroicity in two-dimensional hybrid organic-inorganic perovskites
Source: Nat Commun. 2024 Jul 2;15:5556. doi: 10.1038/s41467-024-49708-w (PMC11220029; doi:10.1038/s41467-024-49708-w)
Supplement: Supplementary file 1 — Supplementary Information [file 41467_2024_49708_MOESM1_ESM.pdf]

Supplementary Information

Chiral Multiferroicity in Two-Dimensional Hybrid Organic-Inorganic Perovskites

Haining Zheng,[1, 2, ‡] Arup Ghosh,[3, ‡] M. J. Swamynadhan,[4, ‡] Qihan Zhang,[5] Walter P. D. Wong,[2] Zhenyue Wu,[2] Rongrong Zhang,[2] Jingsheng Chen,[5] Fanica Cimpoesu,[6] Saurabh Ghosh,[4] Branton J. Campbell,[7] Kai Wang,[8, *] Alessandro Stroppa,[9, *] Ramanathan Mahendiran[3, *] and Kian Ping Loh [1, 2, *]

[1] Joint School of National University of Singapore and Tianjin University, International Campus of Tianjin University, Binhai New City, Fuzhou 350207, China

[2] Department of Chemistry, National University of Singapore, 3 Science Drive 3, Singapore 117543, Singapore

[3] Department of Physics, National University of Singapore, 2 Science Drive 3, Singapore 117551, Singapore

[4] Department of Physics and Nanotechnology, SRM Institute of Science and Technology, Kattankulathur 603203, Tamil Nadu, India

[5] Department of Materials Science and Engineering, National University of Singapore, Singapore 117575, Singapore

[6] Institute of Physical Chemistry, Splaiul Independentei 202, Bucharest 060021, Romania

[7] Department of Physics & Astronomy, Brigham Young University, Provo, UT 84602, United States of America

[8] Key Laboratory of Luminescence and Optical Information, Ministry of Education, School of Physical Science and Engineering and Institute of Optoelectronics Technology, Beijing Jiaotong University, Beijing 100044, China

[9] CNR-SPIN, c/o Dip.to di Scienze Fisiche e Chimiche 67100, Coppito (AQ), Via Vetoio, Italy

Supplementary Note 1. Computational details of constructing the reference structure

The calculation of the ferroelectric polarization in chiral hybrid perovskites deserves special attention, especially for the choice of the reference structure. Since our system is chiral, the reference non-polar structure should be a racemic structure, wherein both (*R*)- and (*S*)-$(MPA)^+$ molecules are simultaneously present in pairs within the unit cell and they are inversion symmetry related. By carefully checking that the inversion center is a unique point in the unit cell common to the organic molecules and framework subsystem, the reference structure is racemic and centrosymmetric.

Constructing a continuously changing transition path from racemic centrosymmetric (non-polar) to chiral (*R*/*S*)-$(MPA)_2CuCl_4$ is complicated since it would involve complete interconversion of (*R*) or (*S*)-MPA molecules to change their chirality (see Supplementary Fig. 13 (b) about the alternative path). Hence, we have constructed a chirality-preserved transition path by introducing a non-centrosymmetric non-polar reference structure by rotating half of the molecules by 180° to compensate for their dipole moments and enforcing the spatial inversion symmetry on the $CuCl_4$ framework[6]. In addition, we also eliminated the Jahn-Teller (J-T) distortions in the inorganic $CuCl_6$ framework, as shown in Fig. 4a. Consequently, we defined a chirality-preserving polarization transition path that develops through roto-displacive distortions. The rotational component of the path acts on the molecules (which are homochiral) while the displacive part acts mainly on the framework, removing the inversion center.

Supplementary Note 2. Calculations on electric dipole moments of organic cations

Since the main contribution to the polarization comes from the organic cations, we calculate the electric dipole moments of the isolated cations, but frozen at their atomic configuration as extracted from the crystal. The polarization arising from the charged organic cation notably depends on the position of the molecular barycenter, whereas that of a neutral molecule would be invariant with respect to this position[5]. Because the dipole moment is proportional to the separation between the barycenter of positive and negative charges, we focus on an intrinsic measure of the isolated MPA^+ cation dipole moment, by estimating it with respect to the barycenter of nuclei positive charges for the molecules. In this approach, one obtains a measure of the "intrinsic" dipole moment of the organic cation, referred to its barycenter of positive charges, while considering the structure of the charged molecules in the crystal and the orientation of the dipole relative to the crystal axes. Having the positive part fixed at zero, the computed dipole moment reveals the relative position of the integrated electronic cloud. This can be taken as a defining feature of the molecule at hand, independent of its spatial repositioning. The orientation of the computed intrinsic MPA^+ dipole moments is drawn with arrows, scaled arbitrarily with respect to the molecular skeleton in Supplementary Fig. 13 (a). This representation includes two different perspective views for both *R* and *S* forms. The molecular dipoles are consistent with a weak ferroelectric arrangement, whose tilting gives rise to a significant in-plane dipole moment, and a small out-of-plane dipole.

Supplementary Note 3. Computational details of exchange parameters and Dzyaloshinsky−Moriya (D-M) interaction

In order to explore the magnetic spin configurations of (*R*/*S*)-$(MPA)_2CuCl_4$ (MPA = *β*-methylphenethylamine), we performed the calculations for the exchange coupling constants (J_{ij}), the D-M antisymmetric exchange interaction parameters (the vector D_i) of neighboring Cu^{2+} ions, and the Single Ion Anisotropy (SIA) parameters, which are all tabulated in Supplementary Table 4. We follow the 4-state method outlined in Refs[1-4]. Our approach considers the classical Heisenberg spin system, which can be expressed as $E = E_0 + E_{spin}$, wherein $E_{spin} = \sum_{(i,j)} J_{ij} S_i . S_j$ and E_0 represents the energy contributed by other interactions. We further analyzed four spin states expressed as follows

$$S_1 = S, S_2 = S \tag{1}$$

$$S_1 = S, S_2 = -S \tag{2}$$

$$S_1 = -S, S_2 = S \tag{3}$$

$$S_1 = -S, S_2 = -S \tag{4}$$

Here S_1 and S_2 refer to the spins of Cu_1 and Cu_2 ions within the intralayer direction, while $S(-S)$ refers to the spins pointing along $a(-a)$, $b(-b)$, and $c(-c)$ directions, respectively. Consequently, it is straightforward to show that these four states give rise to the following energy expressions:

$$E_1 = E_0 + J_{12} S^2 \tag{5}$$

$$E_2 = E_0 - J_{12} S^2 \tag{6}$$

$$E_3 = E_0 - J_{12} S^2 \tag{7}$$

$$E_4 = E_0 + J_{12} S^2 \tag{8}$$

Thus, the magnetic exchange coupling constants J_{12} can be extracted by the following formula, as detailed in Ref.[4]:

$$J_{12} = \frac{E_1 + E_4 - E_2 - E_3}{4S^2} \tag{9}$$

To calculate the SIA parameter, we took into account the influence of spin-orbit coupling (SOC). When the spin exhibits an easy-axis anisotropy, the SIA term can be represented as $H = E_0 + A_1 S^2$. To evaluate the A_1 parameter, we examined six spin states where all spins are aligned along the *a*, -*a*, *b*, -*b*, *c*, and -*c* directions, as described in Ref.[4]. By solving the six equations resulting from these states, we obtained the diagonal SIA terms. Furthermore, we confirmed the magnetic easy-axis direction by pointing the spins along the direction of polarization and slowly rotating them by finite angles.

Regarding the calculation of the D-M interaction parameters, we used the equation $E = E_0 + D_{ij}(S_i \times S_j)$, where D_{ij} represents the D-M vector with *a*, *b*, and *c* components between the ith and j^{th} spins. By aligning the ith and j^{th} spins along the respective *a*- and *b*-axis directions, and vice versa, we derived the D_c terms of D_{ij}. The D_b and D_c terms are calculated using the similar method. The computed values of J_{12}, D_{ij}, and SIA terms are presented in Supplementary Table 4.

Supplementary Note 4. Framework symmetry modes

A symmetry-breaking order parameter of a system is a quantifiable system feature that has a zero amplitude in some higher-symmetry reference state. Such an order parameter often arises from an actual phase transition between the high-symmetry parent and low-symmetry child states having a group-subgroup relationship, though the high-symmetry state may also be hypothetical or physically unrealized.

Crystals grown with purely chiral (*R*/*S*)-MPA^+ molecules have space-group symmetry $P1$ (#1). Though the framework is actually triclinic, it can be viewed as having orthorhombic pseudosymmetry belonging to space group *Cmmm* (#65). This is the topological space group of the framework, meaning that it has no other bond-preserving pseudosymmetries to exploit. In this work, we will use irreducible representations (IRREPs) and order parameter directions (OPDs) of space group *Cmmm* to analyze each of the important distortion modes that deform this idealized parent framework structure into the observed triclinic framework structure. The relevant IRREPs and OPDs appear in Supplementary Table 7, where we employ the IRREP labels[7] and the OPD conventions of the ISODISTORT package of the ISOTROPY Software Suite[8]. Each of the order parameters relevant to $(MPA)_2CuCl_4$ belongs to IRREPs defined at the Γ point $[0,0,0]$ or the R point $[½, ½, ½]$ of the first Brillouin zone. Semicolons are used to separate OPD branches from different arms of the wavevector star, e.g., $[½, ½, ½]$ and $[-½, -½, ½]$ at the R point. Atomic coordinates for the parent framework are shown in Supplementary Table 5.

The system consists of alternating layers of organic chiral-polar (*R*/*S*)-*β*-Methylphenethylamine (MPA^+) molecules and a soft ferromagnetic 2D $CuCl_4$ inorganic framework. Fig. 1a displays the crystal structure of this layered hybrid organic-inorganic perovskite (HOIPs) as determined by single-crystal X-ray diffraction (XRD), which possesses a typical Ruddlesden–Popper configuration such that the layered $[CuCl_4]_2^-$ inorganic octahedral framework is connected via corner-sharing linkages and intercalated by a bilayer of organic cations MPA^+ through N-H···Cl hydrogen bonding,

providing two MPA^+ molecules per Cu atom. Alternating framework layers are offset from one another along a common in-plane crystal direction by half a nearest-neighbor Cu-Cu distance. Each MPA^+ molecule has a chiral tetrahedral center connected to a phenyl group, a methyl group, a methylammonium group, and a hydrogen atom, and has a ferroelectric moment pointing roughly along the C-N bond. Each framework layer experiences a variety of distortions, including in-plane ferroelectric displacements and J-T distortions. The experimental chiral-(*R*) (chiral-(*S*)) framework structures possess a polar triclinic *P*1 space group.

Each $CuCl_4$ octahedron consists of a central Cu atom, two apical Cl1 atoms separated from Cu along parent $\pm a$-axis, two in-plane Cl2 atoms separated from Cu along parent $\pm \boldsymbol{c}$, and two Cl3 atoms separated from Cu along parent $\pm b$-axis. Acting alone, Γ_4^- contributes ferroelectric framework displacements and a net ferroelectric polarization along the parent b-axis. For the framework chiral-(*R*) structure, assuming a stationary Cu position, the action of ferroelectric Γ_4^- causes the pair of apical Cl1 atoms to shift by 0.23 Å along parent $-b$-axis, the in-plane Cl2 atom to shift by 0.28 Å along parent $+\boldsymbol{b}$, and the in-plane Cl3 atom to shift by 0.07 Å along parent $-b$-axis.

As shown in Supplementary Table 7, the distortion mode Γ_4^+, acting alone, enacts a substantial monoclinic shear strain of the crystal lattice around the parent b-axis. It also experiences lattice shear strain and compensating apical-Cl-pair rotations around the same axis belonging to IRREP Γ_4^+.Though this strain mode also shears individual $CuCl_4$ octahedra, Γ_4^+ simultaneously contributes compensating atomic displacements that effectively rotate each pair of apical Cl atoms around the parent b-axis in the same direction, which almost reverses the octahedral distortions. The monoclinic angle of $\beta = 90 + 4.24°$ for the *C*2-symmetry cell of Supplementary Table 5 is quite close to the compensating apical-Cl rotation angle of $\theta = \tan^{-1}\left(\frac{\text{displacement}}{\text{bond length}}\right) = \tan^{-1}\left(\frac{0.1784\ (\text{or}\ 0.1845)\ \text{Å}}{2.3\ \text{Å}}\right) = 4.44^{\circ}$ (or 4.59°) for the chiral-(*R*) or (*S*) structure. When viewed along the parent $+b$-axis direction of the crystal, the compensating Cl rotation

for the chiral-(R) structure occurs in the counterclockwise (CW) sense, so that the compensating axial-vector direction is ferro-rotational (FR) along parent $-b$-axis.

Acting together, the observed ferroelectric (Γ_4^-) and FR (Γ_4^+) order parameters lower the framework symmetry from space group *Cmmm* to *C*2 (#5) with relative conventional basis $\{a, b, c\}$. These are not sufficient for generating the observed triclinic space group and unit cell. The addition of a J-T distortion belonging to two-dimensional IRREP R_1^+ and special OPD $(0; a)$ is required to further lower the symmetry to space group *P*1. Mathematically, any two of $\Gamma_4^-(a)$, $\Gamma_4^+(a)$, and $\Gamma_1^-(a)$ can be superposed to generate the *C*2 symmetry of Supplementary Table 5. However, because Γ_1^- is incapable of directly contributing displacive or occupational modes to the framework-atom Wyckoff sites of *Cmmm*, it cannot serve a primary order parameter of the framework distortion.

The large primary J-T distortion belongs to the two-dimensional IRREP R_1^+ and special OPD $(0; a)$, and is accompanied by two secondary modes of the same wavevector but order-of-magnitude smaller amplitudes. These are an $R_2^+(0; a)$ mode with an antiferro-rotational (AFR) pattern of apical-Cl pair rotations around the in-plane parent -b-axis, which displaces Cl atoms along the in-plane parent c-axis, and an $R_2^-(0; a)$ mode with an antiferroelectric (AFE) pattern of apical-Cl displacements along the parent -b-axis, which could be described as imbalanced octahedral scissor distortions.

If the large observed J-T distortion of the framework were neglected by zeroing all structural contributions from primary IRREP $R_1^+(0; a)$, as well as contributions from the secondary $R_2^+(0; a)$ and $R_2^+(0; a)$ IRREPs, the space-group symmetry of the framework rises to an intermediate level between the parent *Cmmm* and child *P*1 space groups. Whereas the child structure has a symmetry index (ratio of parent-to-child symmetry operation density) of 32, the intermediate space group *C*2 (#5) has a symmetry index of 8 and a conventional unit cell basis matching that of *Cmmm* parent.

Supplementary Note 5. Displacive and occupational interpretations of molecular chirality changes

Though Γ_1^- cannot act on the framework atoms due to their special Wyckoff-site locations within the unit cell, it can act on atoms at general positions (e.g., those of the MPA^+ molecules) and thereby lowers the symmetry from space group *Cmmm* (#65) to *C*222 (#21) when acting alone. Having no mirror planes, space group *C*222 is a Sohncke group and can support chiral structures. Thus, we can view any contribution of Γ_1^- as a chiral order parameter.

Space group $P1$ has sufficient freedom to allow the MPA^+ molecules to be continuously displaced between their *R* to *S* configurations (an effective or 'virtual' mirror operation normal to parent b-axis). Such a displacive order parameter is an alternative to the occupational order parameter described above. Both belong to IRREP Γ_1^- of *Cmmm* and result in the same child structure.

To apply the topological framework symmetry to the MPA^+ molecules as well, one would apply each of the operations of the *Cmmm* parent to the $P1$-symmetry child structure. This would then superpose eight differently oriented copies of MPA^+ at each molecular site (each copy having 12.5% occupancy), four of which would be (*R*)-chiral and four of which would be (*S*)-chiral. Starting from such a high-symmetry molecular-disorder model, one can return to an ordered arrangement of molecules by invoking occupational order parameters belonging to IRREPs Γ_1^- and Γ_4^+ to raise the occupancy of one molecular copy to 100% at each site, while lowering the occupancies of all other copies to zero. The Γ_1^- order parameter simultaneously raises the occupancies of all (*R*)-chiral copies to 100% and lowers the occupancies of all (*S*)-chiral copies to zero, or vice versa, and once it has been imposed so as to obtain a purely chiral molecular configuration, subsequently changing its sign switches the molecular chirality between *R* and *S*. The Γ_4^+ order parameters further resolve amongst four molecular orientations that differ only by proper rotations. We simply recognize that the molecular configuration of MPA^+ in the observed triclinic structures of (*R*)-

$(MPA)_2CuCl_4$ or (*S*)-$(MPA)_2CuCl_4$ is both chiral and ordered, so that the occupational Γ_1^- and Γ_4^+ order parameters are assumed to pre-exist.

Because they belong to the same IRREP, the pre-existing occupational Γ_4^+ order parameter naturally couples to and induces the Γ_4^+ lattice strain and compensating Γ_4^+ apical-Cl-pair rotation of the framework. Similarly, the ferroelectric Γ_4^- molecular displacements couple to and induce ferroelectric Γ_4^- displacements within the framework. However, the chiral Γ_1^- is incapable of acting directly on any of the atoms of $CuCl_4$ framework due to their specific locations (Wyckoff orbits) within the unit cell of parent space group (*Cmmm*). How then can chirality be transferred from the MPA^+ molecules to the framework? This is best understood in terms of the trilinear coupling referred to above, which implies that the superposition of Γ_4^+, and Γ_4^- framework distortions automatically produce a framework structure possessing a chiral space group symmetry, whether or not a chiral order parameter (belonging to Γ_1^-) is active within the framework. Because two non-chiral order parameters combine to produce a chiral structure, one might reasonably refer to this as a “hybrid improper” mechanism of chirality transfer to the framework.

We focus here on a classification and IRREP analysis of the coupling and thereby add insight to the electronic details of the chirality transfer described in the Ref[9]. These ideas can be applied to other 2D HOIP compounds.

Supplementary Note 6. Chirality transfer to the framework and hybrid-improper mechanism

The chiral-(R) and chiral-(S) framework structures can be interchanged via a mirror reflection normal to the parent b-axis. Such a mirror operation reverses the direction of the framework polar moment from the parent $+b$-axis in the chiral-(R) structure to the parent $-b$-axis in the chiral-(S) structure but does not reverse the axial-rotation vector of the apical Cl atoms, which points along parent $-b$-axis in both the chiral-(R) and chiral-(S) structures. Thus, we see that the Γ_4^- polar moment vector $\mathbf{p}$ of the framework and the compensating Γ_4^+ axial FR moment $\mathbf{r}$ of the apical Cl atoms are antiparallel to one another in the chiral-(R) structure but parallel in the chiral-(S) structure. These observations demonstrate that the pseudo-scalar quantity $\xi = \mathbf{p} \cdot \mathbf{r}$ differentiates (R)- and (S)-framework chirality through its sign. The magnitude of ξ further quantifies the extent of the framework chirality through its magnitude; therefore, it appears as an indicator of the chirality transfer in this structure. In the case of racemic structure, constructed by imposing inversion symmetry in the unit cell, the system is centrosymmetric and $\mathbf{p}$ vanishes, hence, $\xi = 0$, consistently with the absence of chirality transfer.

We can see a relative 90° rotation between the MPA^+ molecules on either side of the framework, which results in a large JT framework distortion due to strong N-H···Cl hydrogen bonding to the MPA^+ molecule. The chiral-(R) and chiral-(S) framework structures can be interchanged via a mirror reflection normal to polarization direction (diagonal in ab-plane), as shown in Supplementary Fig. 17. Such a mirror operation reverses the direction of the framework polarization from $[1\ 1\ 0]$ in the chiral-(R) structure to $[\bar{1}\ \bar{1}\ 0]$ in the chiral-(S) structure but does not reverse the axial-rotation vector of the apical Cl atoms in both the chiral-(R) and chiral-(S) structures.

From Fig. 1a, we can see that the chirality-deciding factors are the position of the methyl group and the hydrogen atom and the canting direction of the methylammonium group. The axial rotation of the two apical Cl atoms is due to the interaction of the

framework with the molecule via the C-H-Cl bond between the methyl group and the framework. However, the displacement of the apical Cl atoms is not symmetric around the plane due to the relative 90 angle between either side of the molecules. Hence, the mirror operation which reverses both polarization and chirality keeps the direction of the methyl group and hydrogen unchanged.

An analysis of invariant free-energy polynomials comprised of order parameters of topological space group *Cmmm* reveals a trilinear coupling of Γ_4^+, Γ_4^-, and Γ_1^-. Relative to a *Cmmm* parent with a disordered molecule model, we view a chiral-(*R*) or chiral-(*S*) ordering of the MPA^+ molecules in terms of pre-existing occupational order parameters belonging to IRREPs Γ_1^- and Γ_4^+, which drive the large Γ_4^+ lattice strain and the small compensating FR rotation of the apical-Cl atoms belonging to Γ_4^+. The invariant trilinear coupling term in the free energy then gives rise to a ferroelectric moment belonging to Γ_4^-, which can reasonably be described as a hybrid-improper mechanism.

Supplementary Note 7. Domains of the order parameters

Because the symmetry group of the child structure is a subgroup of that of the parent structure, all symmetry elements of the child also belong to the parent, though many parent symmetry elements are broken or violated by the child structure. The application of any one of these broken parent symmetries to the child structure transforms its order parameters into those of an equivalent domain, where the number of domains (distinct but symmetry-related copies of the child structure) is equal to the index of the subgroup within the parent. In fact, each broken parent symmetry permutes the set of equivalent domains. Each domain of the $P1$-symmetry child of the $Cmmm$-symmetry parent structure of $(MPA)_2CuCl_4$ is listed in Supplementary Table 8, along with a representative parent symmetry operation that realizes it through a transform of the reference domain (#1), and the action of that operation on each of the order parameters described in Supplementary Table 7.

For this structure, 4 distinct lattice-orientation domains of the triclinic lattice are obtained through 180° rotations around the parent a, b, and c axes, in addition to the identity. There are also 4 distinct internal orientation (i.e., lattice preserving) domains obtained via point operations 1, -1, $1'$ and $-1'$, where -1 indicates spatial inversion and $1'$ indicates time reversal. Finally, there are 2 distinct translational domains, one for the identity and one for parent translation $(0, 1, 0)$ or child translation $(\frac{1}{2}, \frac{1}{2}, 0)$. The total number of domains is then $4 \times 4 \times 2 = 32$.

A minus sign indicates a simple reversal of an order parameter. For two or higher-dimensional order parameters, a more complicated transformation of the OPD requires additional explanation. For example, for the J-T distortion, $(0;\, i)$ is the reference domain, $(0; -\, i)$ reverses the long and short axes of each stretched octahedron of the framework, $(-\, i; 0)$ reverses them only in the $x = 0$ layer, and $(i; 0)$ reversed them only in the $x = \frac{1}{2}$ layer.

The experimental structure of (R)-$(MPA)_2CuCl_4$ corresponds to domain #1 (the reference domain) of the order parameters in Supplementary Table 8. Working in another domain may reverse the ferroelectric moment $\mathbf{p}$, the ferrorotational moment $\mathbf{r}$, or the chirality. However, due to the trilinear invariant formed by these three order parameters, if $\xi = +1$ corresponds to an R-chiral structure in one domain, it will do so in all of the domains.

Supplementary Note 8. Possible magneto-electric coupling

Supplementary Table 7 lists the IRREPs and OPDs of topological framework space group $Cmmm$ to which each vector component of the Cu^{2+} electric and magnetic moments. The ferroelectric component $\Gamma_4^-(c)$ along the in-plane parent-Y axis is substantial, whereas the $\Gamma_2^-(d)$ component along the in-plane parent-Z axis and the $\Gamma_3^-(e)$ component along the out-of-plane parent-X axis are either zero or very small. Due to the weak inter-layer magnetic coupling and soft ferromagnetic response, it is appropriate to use ferromagnetic $k = (0,0,0)$ IRREPs to describe the intra-layer ferromagnetic ordering. The A-type antiferromagnetic inter-layer order is actually governed by the corresponding $k = (1,0,0)$ IRREPs which are labeled as Y. This substitution does not affect the free-energy invariants so long as ferro- and anti-ferromagnetic order parameters are not mixed. The in-plane magnetic moment is large, though due to very weak in-plane magnetic anisotropy, that moment can be divided arbitrarily between the $m\Gamma_4^+(f)$ component along the parent-Y axis and the $m\Gamma_2^+(g)$ component along the parent-Z axis. Because the magnetic moment is only weakly canted towards the out-of-plane parent-X axis, the $m\Gamma_3^+(h)$ component is small. Because J-T distortion $R_1^+(0;i)$ is essential for stabilizing the magnetic moments, we can also expect it to play some role in any magneto-electric phenomena.

To explore possible magneto-electric domain switching, all of the unfactorable multi-linear invariants up to order six in these 11 order parameters were identified, each of which is invariant with respect to operations of parent space group $Cmmm$ (i.e., those domain transformations listed in Supplementary Table 8) and cannot be factored into simpler invariant terms. If we select only those multi-linear terms that couple electric and magnetic moments, and ignore terms containing order parameters deemed to be small, an invariant of the form $acfgi^2$ emerges as the most promising candidate. This invariant is linear in the chiral order parameter, the ferroelectric moment, and both in-plane components of the magnetic moment, but quadratic in the amplitude of the J-T distortion. If the $acfgi^2$ term has a substantially non-zero coefficient in the free

energy, and if the magnetic moment has a general in-plane direction, this term could in principle facilitate magneto-electric switching phenomena involving one of the two in-plane components of the magnetic moment. Furthermore, the sign of such a magneto-electric coupling will be opposite for the (*R*) and (*S*)-chiralities. If invariants that involve some of the smaller order parameters are considered, an even richer variety of magneto-electric possibilities emerges.

Supplementary Figures

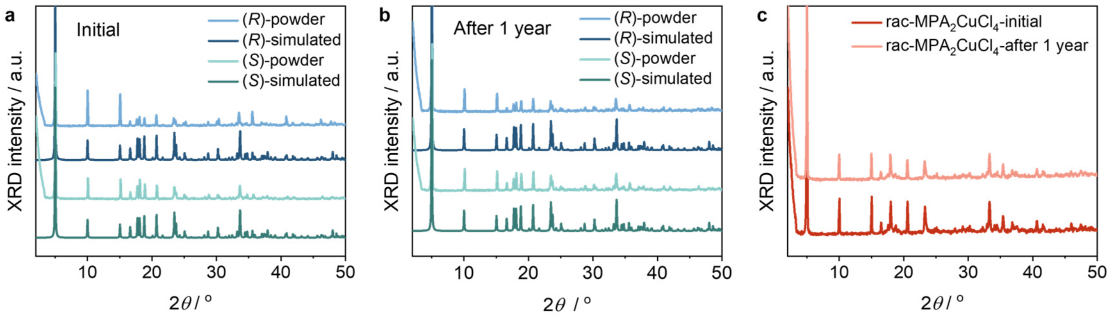

Supplementary Fig. 1. Powder XRD results of chiral copper perovskites to show their long-term stability. (a) The pristine powder XRD results of (*R*/*S*)-$(MPA)_2CuCl_4$ and (b) after one year in an atmospheric environment at room temperature. (c) The pristine powder XRD results of racemic $(MPA)_2CuCl_4$ and after one year in an atmospheric environment at room temperature.

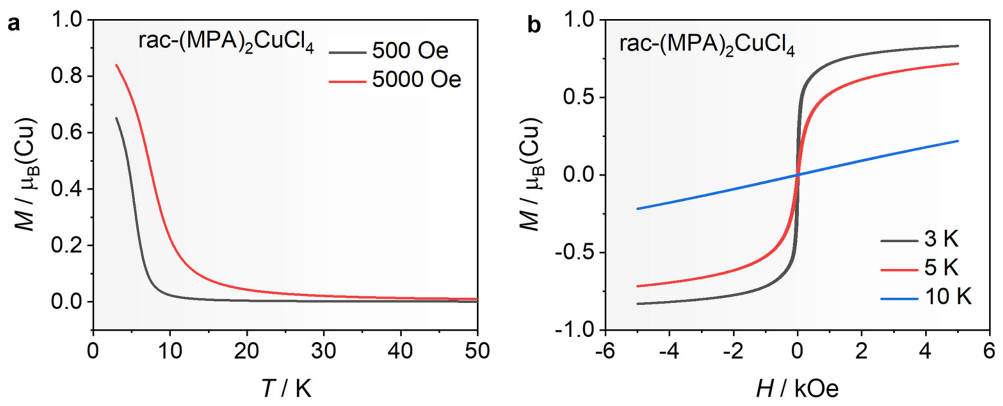

Supplementary Fig. 2. Magnetic characterization of racemic copper perovskites.

(a) Temperature-dependent magnetization (*M*-*T*) curves of racemic $(MPA)_2CuCl_4$ at 500 Oe and 5000 Oe. (b) Field-dependent magnetization (*M*-*H*) curves of racemic $(MPA)_2CuCl_4$ at 3 K, 5 K, and 10 K.

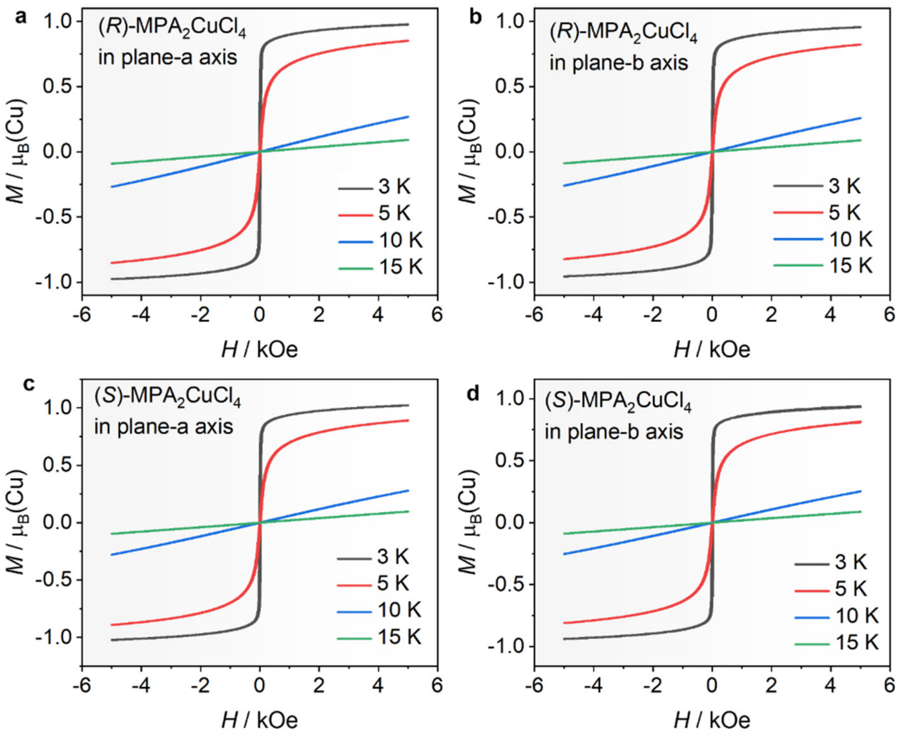

Supplementary Fig. 3. Magnetic characterization of chiral copper perovskites along in-plane directions. (a-b) Field-dependent magnetization (*M*-*H*) curves of (*R*)-$(MPA)_2CuCl_4$ at various temperatures when the magnetic field is along *a*-axis (a) and *b*-axis direction (b). (c-d) Field-dependent magnetization (*M*-*H*) curves of (*S*)-$(MPA)_2CuCl_4$ at various temperatures when the magnetic field is along *a*-axis (c) and *b*-axis direction (d).

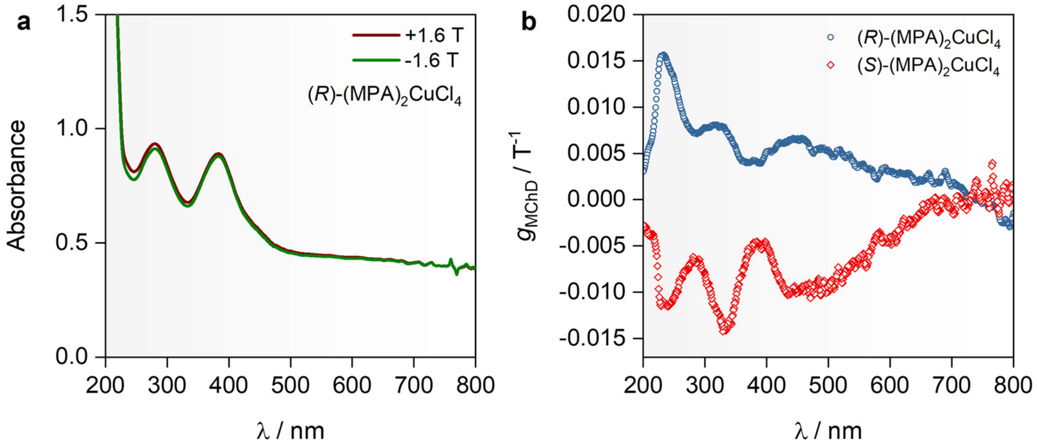

Supplementary Fig. 4. Field-dependent absorbance spectra and the corresponding dissymmetry factor. (a) Field-dependent absorbance spectra of (*R*)- and (*S*)-$(MPA)_2CuCl_4$ at room temperature. (b) The dissymmetry factor of magneto-chiral dichroism (g_{MChD}) for (*R*)- and (*S*)-$(MPA)_2CuCl_4$. All the measurements were conducted in ambient conditions.

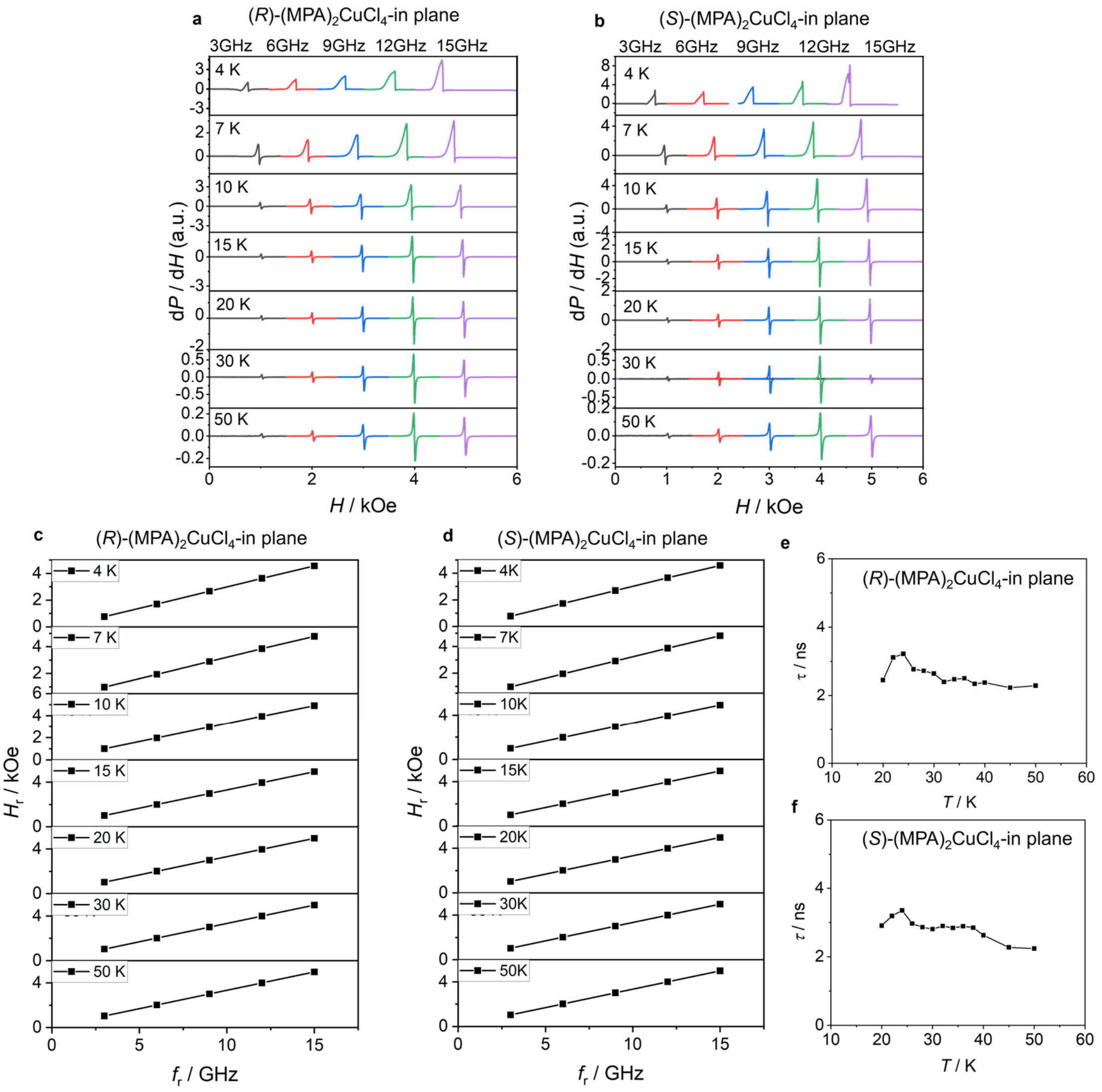

Supplementary Fig. 5. Ferromagnetic resonance (FMR) spectra of chiral copper perovskites along the in-plane direction. (a-b) Field-dependent FMR spectra for (*R*)-$(MPA)_2CuCl_4$ (a) and (*S*)-$(MPA)_2CuCl_4$ (b) at various temperatures when the magnetic field is applied along the in-plane direction. (c-d) Frequency (f_r) dependence of resonance field (H_r) for (*R*)-$(MPA)_2CuCl_4$ (c) and (*S*)-$(MPA)_2CuCl_4$ (d) along the in-plane direction. (e-f) Temperature-dependent spin decoherence lifetime τ for (*R*)-$(MPA)_2CuCl_4$ (e) and (*R*)-$(MPA)_2CuCl_4$ (f).

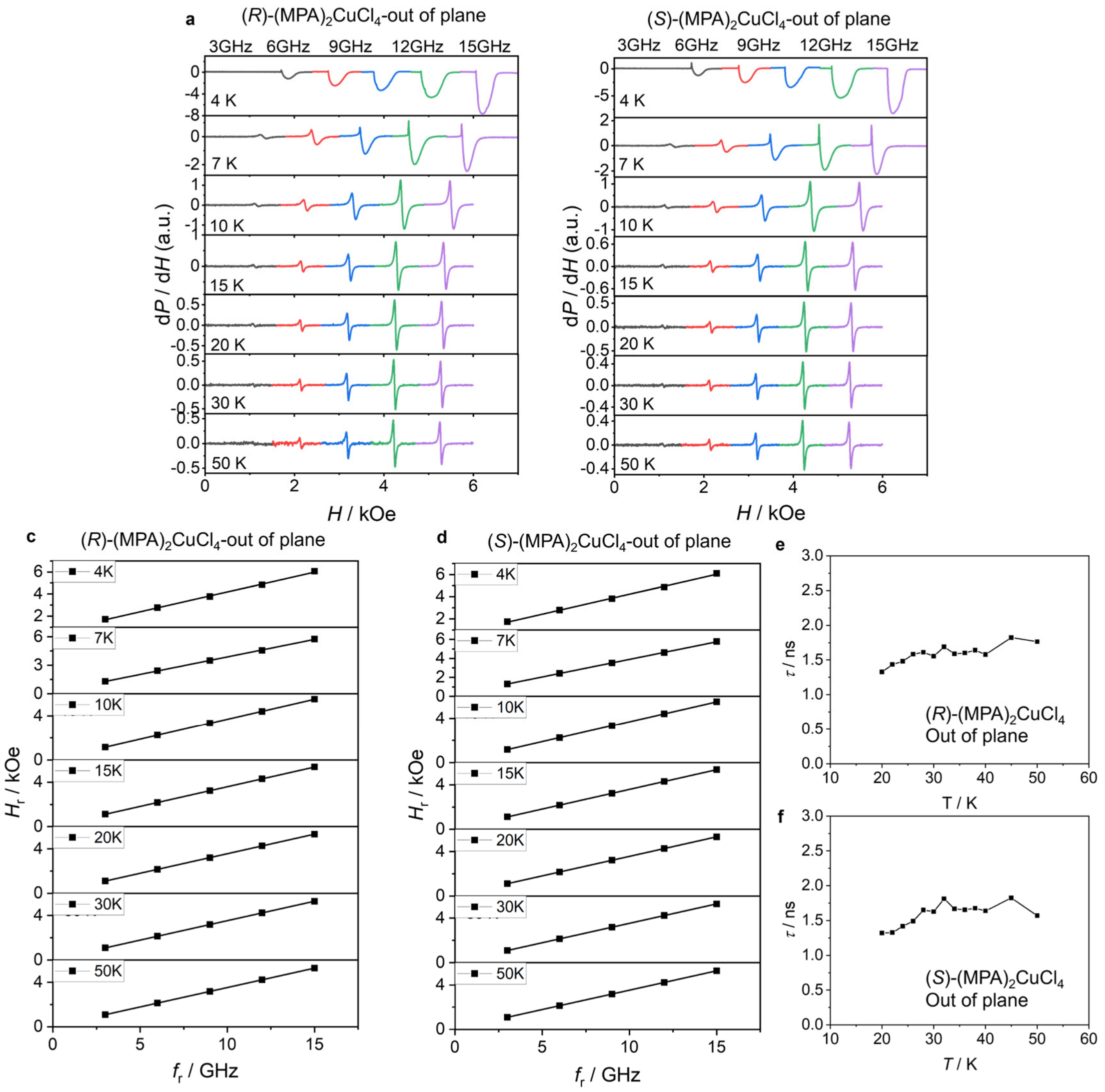

Supplementary Fig. 6. FMR spectra of chiral copper perovskites along the out-of-plane direction. (a-b) Field-dependent FMR spectra for (*R*)-$(MPA)_2CuCl_4$ (a) and (*R*)-$(MPA)_2CuCl_4$ (b) at various temperatures when the magnetic field is applied along the out-of-plane direction. (c-d) Frequency (f_r) dependence of resonance field (H_r) for (*R*)-$(MPA)_2CuCl_4$ (a) and (*S*)-$(MPA)_2CuCl_4$ (d) along the out-of-plane direction. (e-f) Temperature-dependent spin decoherence lifetime τ for (*R*)-$(MPA)_2CuCl_4$ (e) and (*R*)-$(MPA)_2CuCl_4$ (f).

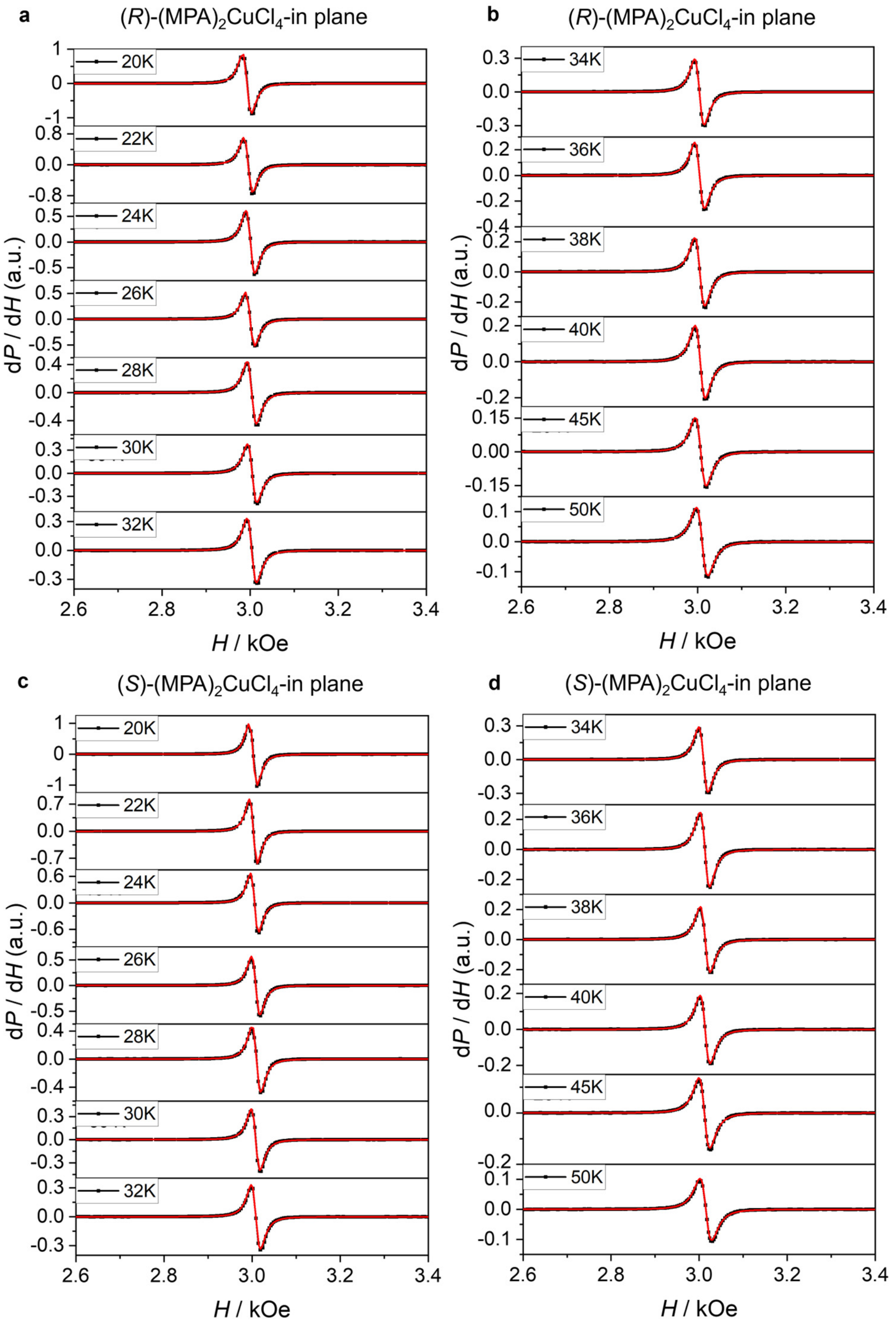

Supplementary Fig. 7. Field-dependent FMR spectra at paramagnetic regimes and corresponding Lorentzian fitting along the in-plane direction. FMR and Lorentzian fitting (red line) from 20 K to 50 K for (*R*)- $(MPA)_2CuCl_4$ (a-b) and (*S*)-$(MPA)_2CuCl_4$ (c-d).

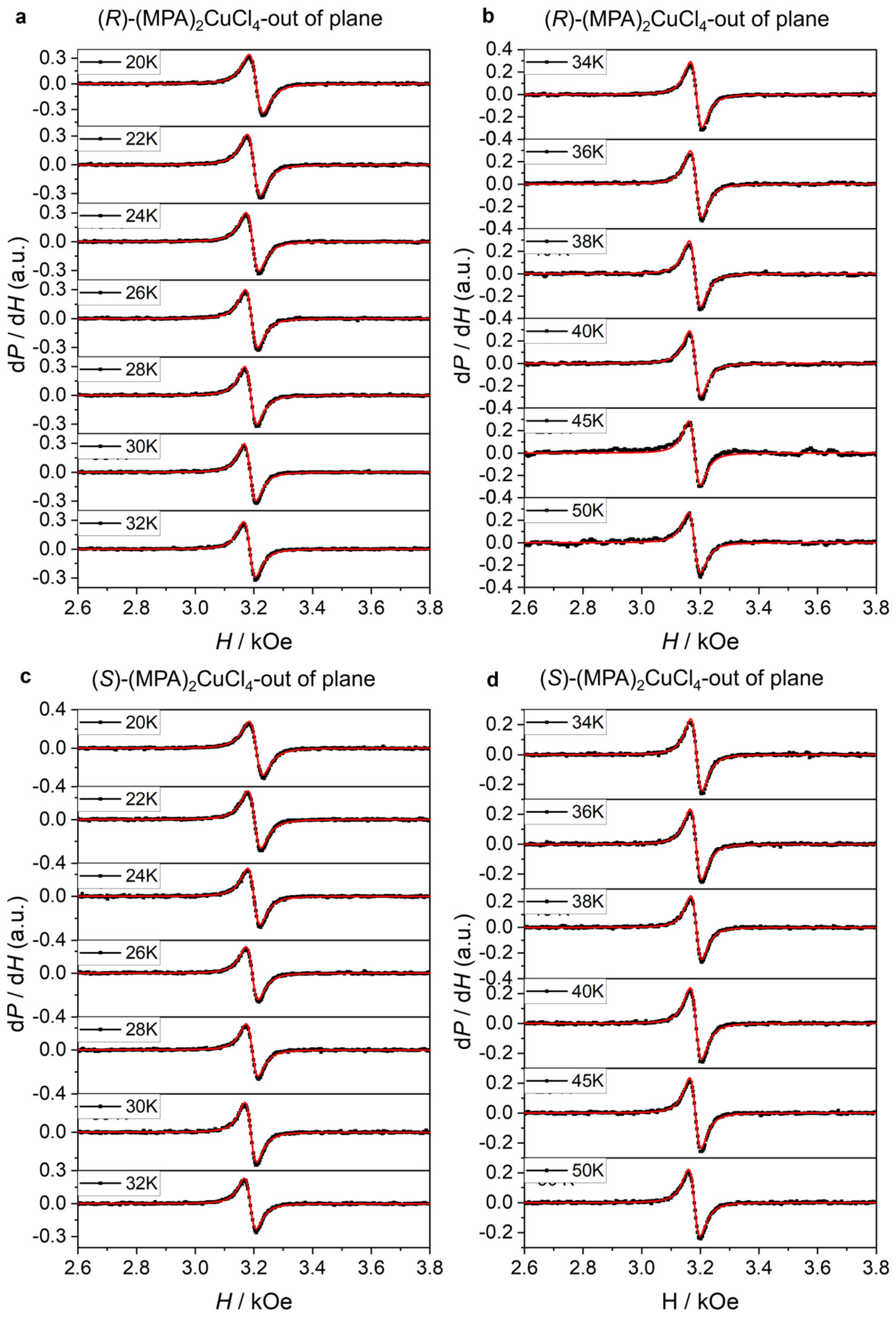

Supplementary Fig. 8. Field-dependent FMR spectra at paramagnetic regimes and corresponding Lorentzian fitting along the out-of-plane direction. FMR and Lorentzian fitting (red line) from 20 K to 50 K for (*R*)- $(MPA)_2CuCl_4$ (a-b) and (*S*)-$(MPA)_2CuCl_4$ (c-d).

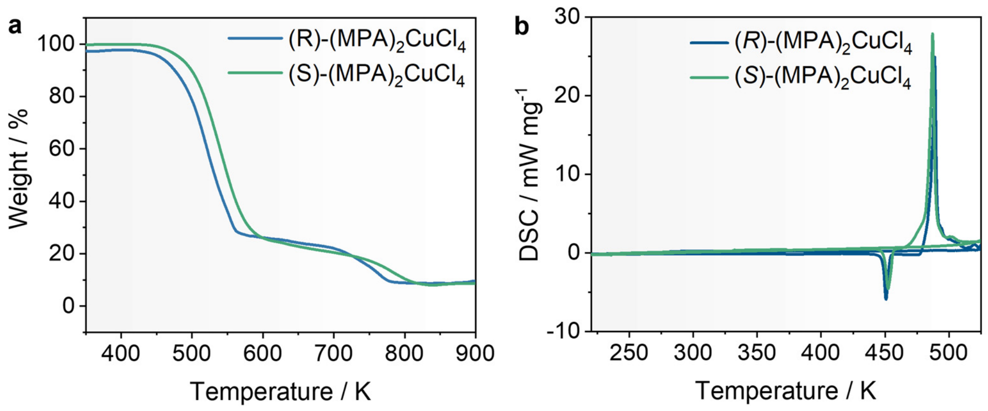

Supplementary Fig. 9. Thermostability and decomposition temperatures for chiral copper perovskites. (a) Thermogravimetric analysis (TGA) curves of (*R*/*S*)-$(MPA)_2CuCl_4$. (b) Differential scanning calorimetry (DSC) curves of (*R*/*S*)-$(MPA)_2CuCl_4$.

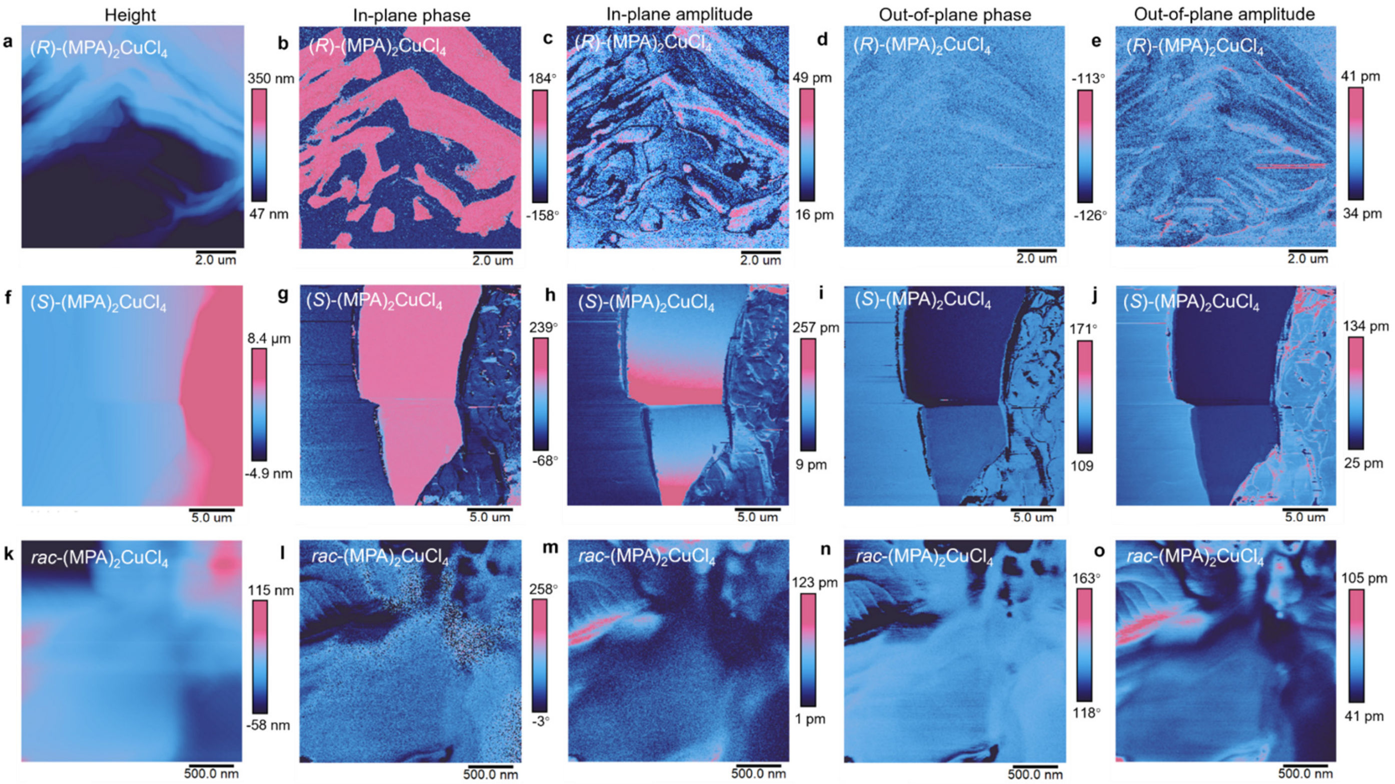

Supplementary Fig. 10. Piezoresponse force microscopy (PFM) characterization for chiral and racemic copper perovskites. PFM images of height (a, f, k), in-plane phase (b, g, l), in-plane amplitude (c, h, m), out-of-plane phase (d, i, n) and out-of-plane amplitude (e, j, o) for (*R*/*S*/racemic)-$(MPA)_2CuCl_4$.

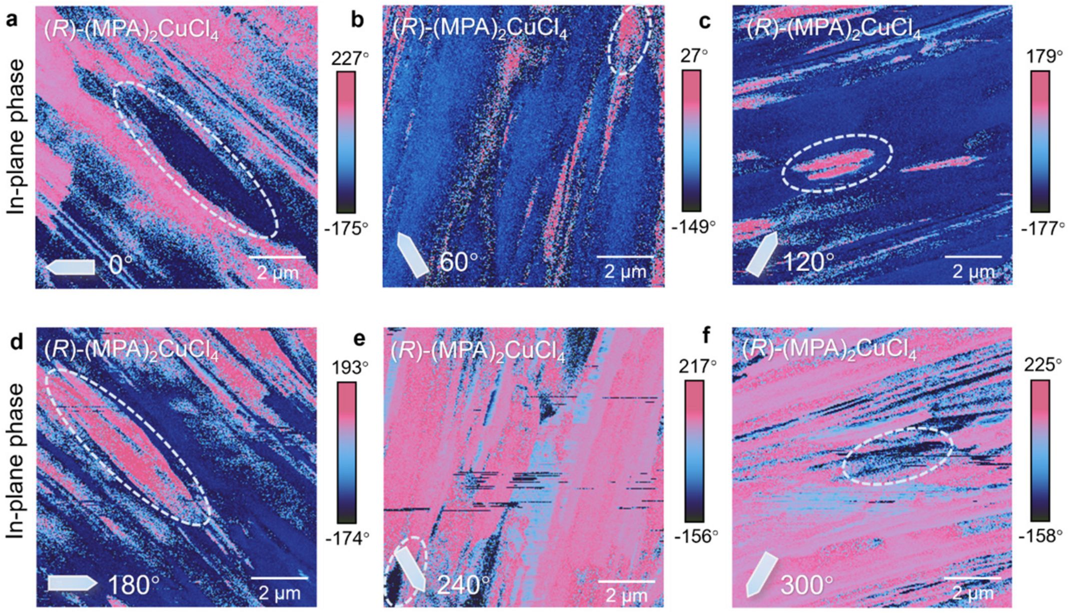

Supplementary Fig. 11. PFM images to show the angular dependence of domain evolution for (*R*)-$(MPA)_2CuCl_4$. In-plane PFM phase images of (*R*)-$(MPA)_2CuCl_4$ with the relative sample-PFM tip angle of 0° (a), 60° (b), 120° (c), 180° (d), 240° (e), 300° (f). The white broken circles highlight the opposite contrast of in-plane ferroelectric domains when the angular difference is 180°.

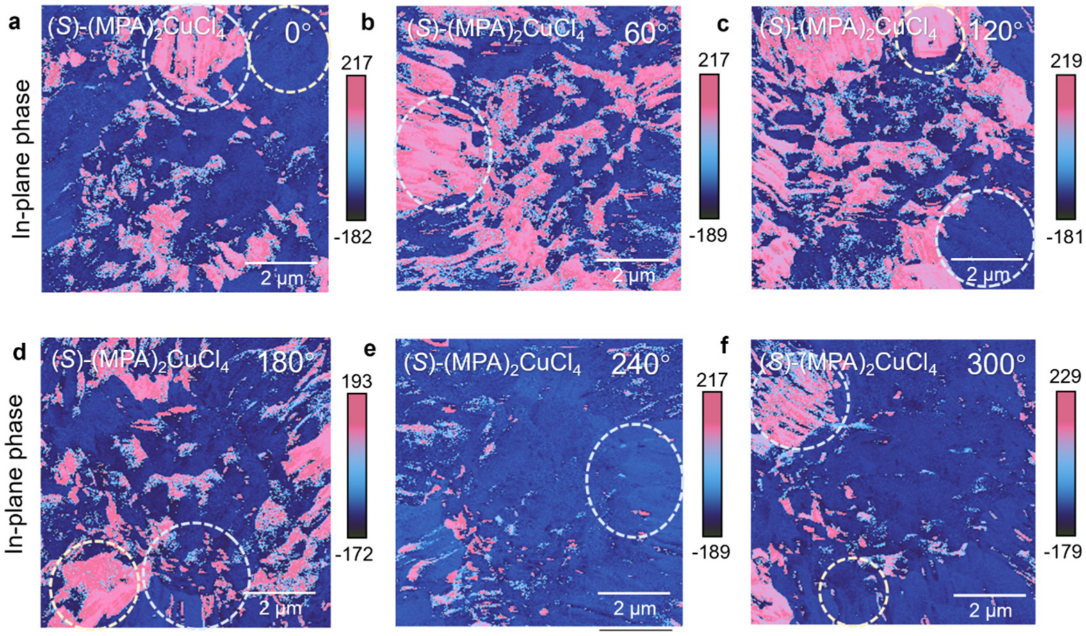

Supplementary Fig. 12. PFM images to show the angular dependence of domain evolution for (*S*)-(MPA)$_2$CuCl$_4$. Angle-dependent PFM in-plane images of (*S*)-(MPA)$_2$CuCl$_4$ with the relative sample-PFM tip angles of 0° (a), 60° (b), 120° (c), 180° (d), 240° (e) and 300° (f). The white broken circles highlight the opposite contrast of in-plane ferroelectric domains when the angular difference is 180°.

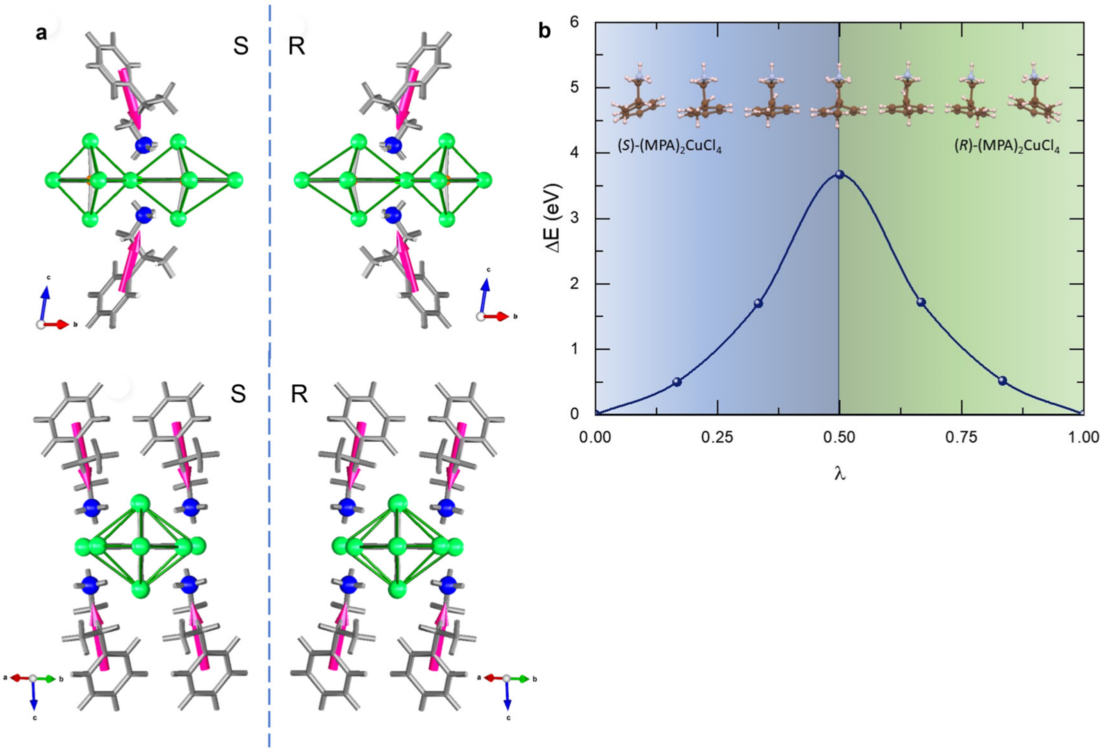

Supplementary Fig. 13. Calculation results of electric dipole moments of organic cations. (a) Crystal structures of (*S*)- or (*R*)-$(MPA)_2CuCl_4$ showing the orientation of MPA^+ dipole moments (red arrows). (b) Alternative path that switches from (*S*)-$(MPA)_2CuCl_4$ to (*R*)-$(MPA)_2CuCl_4$. Here the lowest possible path that changes from (*S*)-$(MPA)_2CuCl_4$ to (*R*)-$(MPA)_2CuCl_4$ is shown, where the molecule is distorted, and it crosses a 'virtual mirror' for changing the chirality. The brown, white, grey, blue, and green atoms represent carbon, hydrogen, nitrogen, copper, and chlorine, respectively.

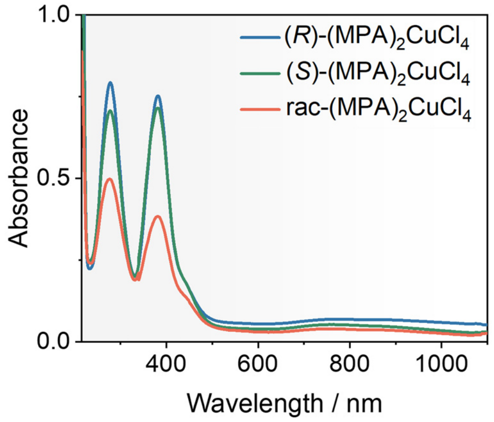

Supplementary Fig. 14. Ultraviolet−visible−near-infrared spectra for (*R*/*S*/racemic)-$(MPA)_2CuCl_4$.

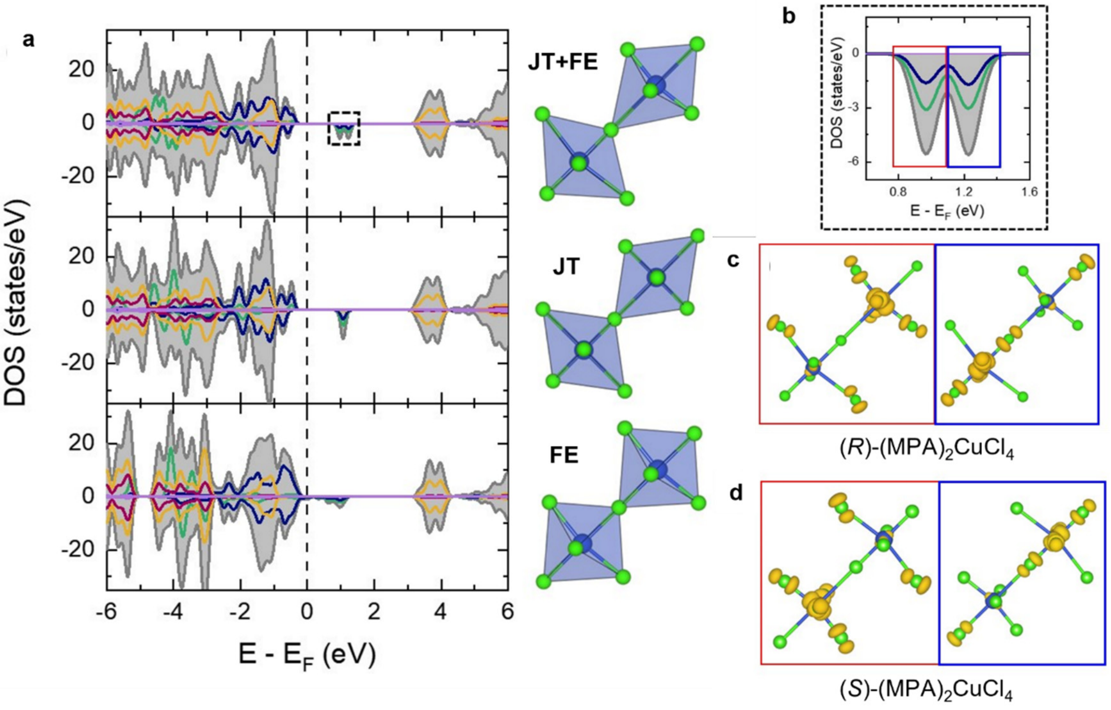

Supplementary Fig. 15. Calculation results of the density of states (DOS) and partial charge densities. Effects of J-T distortion and ferroelectric distortion on the electronic structure. (a) DOS showing the ground-state structure with both J-T and ferroelectric distortion (top), the structure with only a J-T distortion and no ferroelectric distortion (middle), and the structure with only a ferroelectric distortion (bottom), with the respective frameworks viewed along the crystallographic *c*-axis. (b) Enlarged image of the states that belong to two inequivalent Cu atom sites, with their respective partial charge densities shown in (c) and (d).

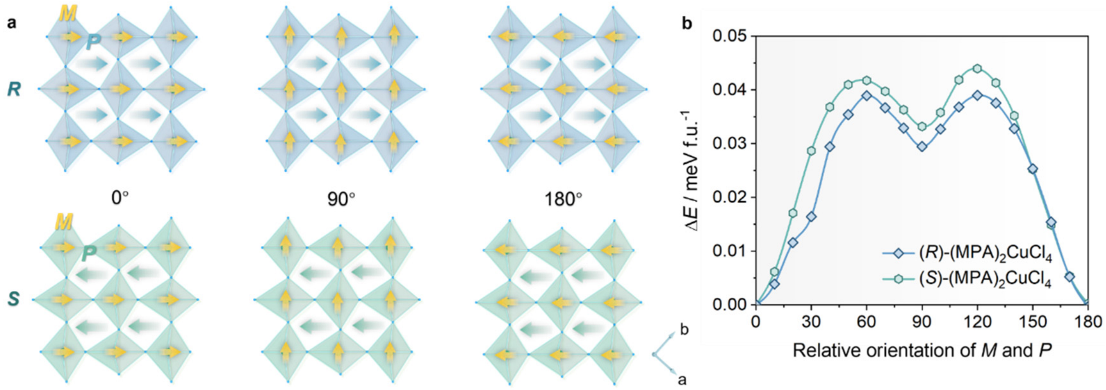

Supplementary Fig. 16. Simulations on magnetic moment *M* with respect to the polarization direction *P*. (a) Schematic showing the parallel (0°), perpendicular (90°), and anti-parallel orientation (180°) of spin (*M*, blue arrows) with respect to the polarization direction (*P*, yellow arrows) for (*R*)- (top) and (*S*)-$(MPA)_2CuCl_4$ (bottom). (b) Energy evolution as the relative orientation of *M* and *P* varies from 0° to 180° for (*R*/*S*)-$(MPA)_2CuCl_4$.

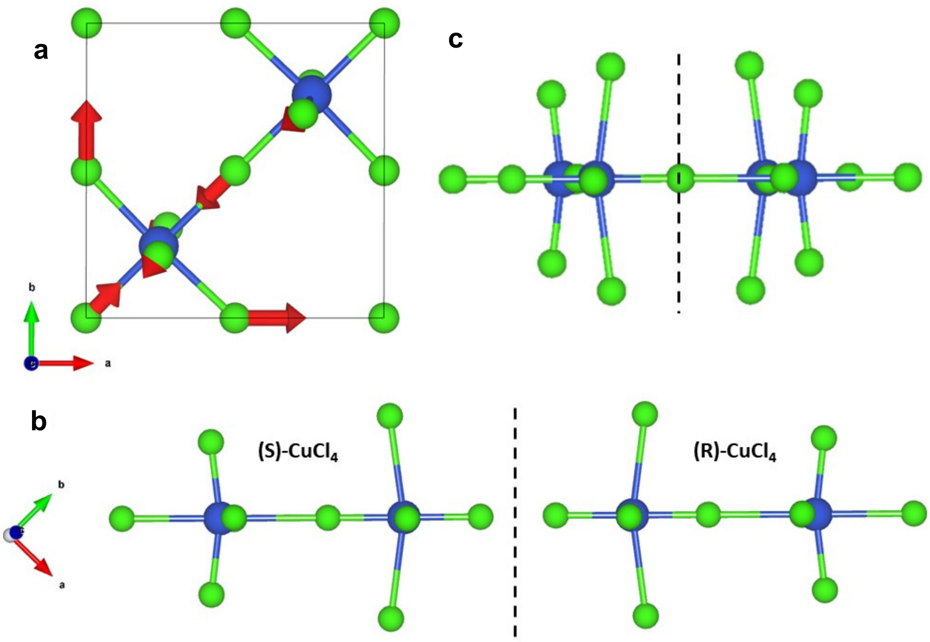

Supplementary Fig. 17. Schematic diagram demonstrating the J-T distortion and chirality transferred to the framework. (a) The primary J-T mode, ferroelectric distortion, and compensating apical-Cl-pair rotations acting on the in-plane and apical Cl atoms. (b) Comparison of the (*S*)-chiral and (*R*)-chiral framework distortions, which are interchanged by an improper mirror plane (the dashed line), but not by any proper rotation. (c) (*S*)-chiral and (*R*)-chiral framework connected at the central Cl atom through which the mirror plane passes.

Supplementary Tables

**(*R*)-$(MPA)_2CuCl_4$**				
Empirical formula	$C_{18}H_{28}Cl_4CuN_2$	$C_{18}H_{28}Cl_4CuN_2$	$C_{18}H_{28}Cl_4CuN_2$	$C_{18}H_{28}Cl_4CuN_2$
Formula weight	477.76 g/mol	477.76 g/mol	477.76 g/mol	477.76 g/mol
Temperature/K	100.00 K	150.00 K	200.00 K	300.00 K
Crystal system	triclinic	triclinic	triclinic	triclinic
Space group	*P* 1	*P* 1	*P* 1	*P* 1
a/Å	7.5711(3) Å	7.5916(4) Å	7.6137(3) Å	7.6655(3) Å
b/Å	7.5732(3) Å	7.5949(3) Å	7.6180(2) Å	7.6684(3) Å
c/Å	17.8700(7) Å	17.8982(9) Å	17.9298(6) Å	17.9969(7) Å
α/°	80.8830(10)°	80.926(2)°	80.9850(10)°	81.1260(10)°
β/°	86.802(2)°	86.753(2)°	86.6570(10)°	86.4910(10)°
γ/°	89.4090(10)°	89.3770(10)°	89.3960(10)°	89.4380(10)°
Volume/$Å^3$	1010.10(7) $Å^3$	1017.41(8) $Å^3$	1025.35(6) $Å^3$	1043.27(7) $Å^3$
Z	2	2	2	2
Density (calculated)	1.571 g/cm^3	1.560 g/cm^3	1.547 g/cm^3	1.521 g/cm^3
Absorption coefficient	1.615 mm^{-1}	1.603 mm^{-1}	1.591 mm^{-1}	1.563 mm^{-1}
F(000)	494.0	494.0	494.0	494.0
2Θ range for data collection/°	4.624 to 61.066	4.616 to 61.024	4.608 to 61.122	4.59 to 61.06
Reflections collected	56229	54074	55860	54316
Data/restraints/parameters	12271/95/528	12336/95/528	12490/95/528	12700/95/528
Goodness-of-fit on F^2	1.043	1.021	1.033	1.030
Final R indexes [I>=2σ (I)]	R_1 = 0.0270, wR_2 = 0.0594	R_1 = 0.0258, wR_2 = 0.0594	R_1 = 0.0294, wR_2 = 0.0628	R_1 = 0.0355, wR_2 = 0.0747
Flack parameter	0.001(4)	-0.003(3)	-0.003(4)	-0.002(5)

Supplementary Table 1. Single-crystal XRD results for (*R*)-$(MPA)_2CuCl_4$.

**(*S*)-$(MPA)_2CuCl_4$**				
Empirical formula	$C_{18}H_{28}Cl_4CuN_2$	$C_{18}H_{28}Cl_4CuN_2$	$C_{18}H_{28}Cl_4CuN_2$	$C_{18}H_{28}Cl_4CuN_2$
Formula weight	477.76 g/mol	477.76 g/mol	477.76 g/mol	477.76 g/mol
Temperature/K	100.00 K	150.00 K	200.00 K	300.00 K
Crystal system	triclinic	triclinic	triclinic	triclinic
Space group	*P* 1	*P* 1	*P* 1	*P* 1
a/Å	7.5876(3) Å	7.5917(3) Å	7.6164(2) Å	7.6652(3) Å
b/Å	7.5928(3) Å	7.5934(3) Å	7.6171(3) Å	7.6741(2) Å
c/Å	17.9130(7) Å	17.8945(7) Å	17.9279(6) Å	18.0025(7) Å
α/°	80.8960(10)°	80.9730(10)°	81.0150(10)°	81.1800(10)°
β/°	86.8300(10)°	86.7510(10)°	86.6890(10)°	86.532(2)°
γ/°	89.3750(10)°	89.3910(10)°	89.3970(10)°	89.4260(10)°
Volume/$Å^3$	1017.43(7) $Å^3$	1017.15(7) $Å^3$	1025.61(6) $Å^3$	1044.53(6) $Å^3$
Z	1	2	2	2
Density (calculated)	1.560 g/cm^3	1.560 g/cm^3	1.547 g/cm^3	1.519 g/cm^3
Absorption coefficient	1.603 mm^{-1}	1.604 mm^{-1}	1.590 mm^{-1}	1.562 mm^{-1}
F(000)	494.0	494.0	494.0	494.0
2Θ range for data collection/°	4.612 to 60.974°	4.616 to 61.012°	4.608 to 61.008°	4.588 to 61.004°
Reflections collected	66898	74460	77766	59409
Data/restraints/parameters	12244/95/504	12367/95/504	12466/94/504	12714/94/504
Goodness-of-fit on F^2	1.042	1.024	1.046	1.037
Final R indexes [I>=2σ (I)]	R_1 = 0.0274, wR_2 = 0.0595	R_1 = 0.0308, wR_2 = 0.0644	R_1 = 0.0298, wR_2 = 0.0614	R_1 = 0.0384, wR_2 = 0.0772
Flack parameter	-0.010(3)	-0.004(4)	-0.003(4)	-0.003(6)

Supplementary Table 2. Single-crystal XRD results for (*S*)-$(MPA)_2CuCl_4$.

**λ (nm)**	**230**	**280**	**330**	**390**	**430**
**(R)-$(MPA)_2CuCl_4$/g_{MChD} ($\times 10^{-3}$ T^{-1})**	16	7	8	4	6
**(S)-$(MPA)_2CuCl_4$/g_{MChD} ($\times 10^{-3}$ T^{-1})**	11	7	14	5	10

Supplementary Table 3. Extracted absolute values of g_{MChD} from the Supplementary Fig. 4 at specific wavelengths.

	J_{ab} (meV)	D_a (meV)	D_b (meV)	D_c (meV)	$\lvert D \rvert$ (meV)	SIA (meV)
(*R*)-$(MPA)_2CuCl_4$	5.5	2.2	0.4	0.4	2.3	0.2
(*S*)-$(MPA)_2CuCl_4$	5.7	0.8	2.3	-0.7	2.6	0.2

Supplementary Table 4. Summary of calculation results of J_{ab}, D_i vectors and SIA parameters.

SG	$\boldsymbol{a}$	$\boldsymbol{b}$	$\boldsymbol{c}$	$\boldsymbol{\alpha}$	$\boldsymbol{\beta}$	$\boldsymbol{\gamma}$	Relative conv. basis
$Cmmm$	35.3838	5.3600	5.3600	90	90	90	$\{a, b, c\}$
$C2$	35.3838	5.3908	5.3336	90	94.237	90	$\{a, b, c\}$
$P1$ (R)	7.5824	7.5873	17.8989	80.866	86.869	89.384	$\{b - c, b + c, ½(a + b)\}$
$P1$ (S)	7.5840	7.5825	17.9024	80.844	86.806	89.389	$\{b - c, b + c, ½(a + b)\}$

Supplementary Table 5. Unit cell parameters and relative bases of the topological framework structure (space group *Cmmm*), the experimental chiral-(*R*) and chiral-(*S*) framework structures (space group *P*1), and corresponding hypothetical chiral framework structures (space group *C*2) obtained by eliminating the large observed J-T distortions. Each of these cells has approximately the same conventional cell volume (~ 1015 $Å^3$). The in-plane cell parameter of the *Cmmm* structure was chosen to be approximately half-way between those of the *P*1 structures.

Atom	Multiplicity	Wyckoff	Position	Framework component
Cu	2	a	(0,0,0)	center
Cl1	4	g	(-0.06396,0,0)	apical halide
Cl2	2	d	(0,0,1/2)	in-plane shared halide
Cl3	2	b	(1/2,0,0)	in-plane shared halide

Supplementary Table 6. Unit cell parameters and relative bases of the topological framework structure (space group *Cmmm*).

IRREP	OPD	(M)SG	Relative basis	OP name	OP description
Γ_1^-	(a)	$C222$, #21	$\{a, b, c\}$	chiral	Molecular chirality
Γ_4^+	(b)	$C2/m$, #12	$\{a, b, c\}$	FR - parent$Y$	Large monoclinic strain and small apical-Cl ferro-rotation
Γ_4^-	(c)	$Amm2$, #38	$\{c, a, b\}$	FE - parent$Y$	Modest in-plane FE displacement
Γ_2^-	(d)	$Cmm2$, #35	$\{a, b, c\}$	FE - parent$Z$	Very small in-plane FE displacement
Γ_3^-	(e)	$Amm2$, #38	$\{c, b, -a\}$	FE - parent$X$	Very small out-of-plane FE displacement
mΓ_4^+	(f)	$Cmm'm'$ #65.486	$\{b, a, -c\}$	FM - parent$Y$	Large isotropic in-plane FM moment
mΓ_2^+	(g)	$Cm'm'm$ #65.485	$\{a, b, c\}$	FM - parent$Z$	Potentially large isotropic in-plane FM moment
mΓ_3^+	(h)	$Cmm'm'$ #65.486	$\{a, b, c\}$	FM - parent$X$	Small out-of-plane FM moment
R_1^+	$(0; i)$	$C2/m$, #12	$\{a-b, 2c, -½(a+b)\}$	AFE - J-T	Large in-plane J-T distortion
R_2^+	$(0; j)$	$C2/m$, #12	$\{a-b, 2c, -½(a+b)\}$	AFR - parent$Y$	Small imbalanced apical-Cl AFRs
R_2^-	$(0; k)$	$C2/m$, #12	$\{a-b, 2c, -½(a+b)\}$	AFE - parent$Y$	Small apical-Cl scissor distortion

Supplementary Table 7. Summary of mode analysis. For each order parameter observed or relevant to the framework of $(MPA)_2CuCl_4$, we list the corresponding IRREP and OPD of space group *Cmmm*, the symmetry group, and the relative basis that results when it acts alone, a short name that conveys tensor type and orientation, and a brief description of the mode pattern. For a magnetic order parameter, a UNI symbol and BNS number were employed to convey the resulting magnetic space group (MSG). The abbreviations FE, FR, FM, AFE, AFR represent ferroelectric, ferro-rotational, ferromagnetic, antiferroelectric, and antiferro-rotational, respectively.

ID	Parent symmetry	Indices	Action on order parameters
#01	(x,y,z)	1 1 1	(a| b| c| d| e| f| g| h| 0; i| 0; j| 0; k)
#17	(x,y-1,z)	1 1 2	(a| b| c| d| e| f| g| h| 0;-i| 0;-j| 0;-k)
#02	(-x,-y,-z)	1 2 1	(-a| b|-c|-d|-e| f| g| h| 0; i| 0; j| 0;-k)
#18	(-x,-y+1,-z)	1 2 2	(-a| b|-c|-d|-e| f| g| h| 0;-i| 0;-j| 0; k)
#03	(x,y,z)'	1 3 1	(a| b| c| d| e|-f|-g|-h| 0; i| 0; j| 0; k)
#19	(x,y-1,z)'	1 3 2	(a| b| c| d| e|-f|-g|-h| 0;-i| 0;-j| 0;-k)
#04	(-x,-y,-z)'	1 4 1	(-a| b|-c|-d|-e|-f|-g|-h| 0; i| 0; j| 0;-k)
#20	(-x,-y+1,-z)'	1 4 2	(-a| b|-c|-d|-e|-f|-g|-h| 0;-i| 0;-j| 0; k)
#05	(x,-y,-z)	2 1 1	(a|-b|-c|-d| e|-f|-g| h| i; 0| j; 0| k; 0)
#21	(x,-y+1,-z)	2 1 2	(a|-b|-c|-d| e|-f|-g| h|-i; 0|-j; 0|-k; 0)
#06	(-x,y,z)	2 2 1	(-a|-b| c| d|-e|-f|-g| h| i; 0| j; 0|-k; 0)
#22	(-x,y-1,z)	2 2 2	(-a|-b| c| d|-e|-f|-g| h|-i; 0|-j; 0| k; 0)
#07	(x,-y,-z)'	2 3 1	(a|-b|-c|-d| e| f| g|-h| i; 0| j; 0| k; 0)
#23	(x,-y+1,-z)'	2 3 2	(a|-b|-c|-d| e| f| g|-h|-i; 0|-j; 0|-k; 0)
#08	(-x,y,z)'	2 4 1	(-a|-b| c| d|-e| f| g|-h| i; 0| j; 0|-k; 0)
#24	(-x,y-1,z)'	2 4 2	(-a|-b| c| d|-e| f| g|-h|-i; 0|-j; 0| k; 0)
#09	(-x,y,-z)	3 1 1	(a| b| c|-d|-e| f|-g|-h| i; 0|-j; 0|-k; 0)
#25	(-x,y-1,-z)	3 1 2	(a| b| c|-d|-e| f|-g|-h|-i; 0| j; 0| k; 0)
#10	(x,-y,z)	3 2 1	(-a| b|-c| d| e| f|-g|-h| i; 0|-j; 0| k; 0)
#26	(x,-y+1,z)	3 2 2	(-a| b|-c| d| e| f|-g|-h|-i; 0| j; 0|-k; 0)
#11	(-x,y,-z)'	3 3 1	(a| b| c|-d|-e|-f| g| h| i; 0|-j; 0|-k; 0)
#27	(-x,y-1,-z)'	3 3 2	(a| b| c|-d|-e|-f| g| h|-i; 0| j; 0| k; 0)
#12	(x,-y,z)'	3 4 1	(-a| b|-c| d| e|-f| g| h| i; 0|-j; 0| k; 0)
#28	(x,-y+1,z)'	3 4 2	(-a| b|-c| d| e|-f| g| h|-i; 0| j; 0|-k; 0)
#13	(-x,-y,z)	4 1 1	(a|-b|-c| d|-e|-f| g|-h| 0; i| 0;-j| 0;-k)
#29	(-x,-y+1,z)	4 1 2	(a|-b|-c| d|-e|-f| g|-h| 0;-i| 0; j| 0; k)
#14	(x,y,-z)	4 2 1	(-a|-b| c|-d| e|-f| g|-h| 0; i| 0;-j| 0; k)
#30	(x,y-1,-z)	4 2 2	(-a|-b| c|-d| e|-f| g|-h| 0;-i| 0; j| 0;-k)
#15	(-x,-y,z)'	4 3 1	(a|-b|-c| d|-e| f|-g| h| 0; i| 0;-j| 0;-k)
#31	(-x,-y+1,z)'	4 3 2	(a|-b|-c| d|-e| f|-g| h| 0;-i| 0; j| 0; k)

#16	(x,y,-z)'	4 4 1	(-a|-b| c|-d| e| f|-g| h| 0; i| 0;-j| 0; k)
#32	(x,y-1,-z)'	4 4 2	(-a|-b| c|-d| e| f|-g| h| 0;-i| 0; j| 0;-k)

Supplementary Table 8. Domains of the *P*1-symmetry child of the *Cmmm*-symmetry parent, as generated by ISODISTORT. For each domain, we list (1) a unique sequential identifier (used by ISODISTORT), (2) a representative parent symmetry operation (primes indicate magnetic time reversal), (3) three integer indices that convey the decomposition of the symmetry operation into a lattice-reorientation, a lattice-preserving point operation, and a translation, and (4) the action of the symmetry operation on each of the order parameters discussed in Supplementary Table 7.

References

1 Whangbo, M.-H., Koo, H.-J. & Dai, D. Spin exchange interactions and magnetic structures of extended magnetic solids with localized spins: Theoretical descriptions on formal, quantitative and qualitative levels. *J. Solid State Chem.* **176**, 417-481 (2003).

2 Xiang, H. J., Kan, E. J., Wei, S.-H., Whangbo, M. H. & Gong, X. G. Predicting the spin-lattice order of frustrated systems from first principles. *Phys. Rev. B* **84**, 224429 (2011).

3 Weingart, C., Spaldin, N. & Bousquet, E. Noncollinear magnetism and single-ion anisotropy in multiferroic perovskites. *Phys. Rev. B* **86**, 094413 (2012).

4 Li, X. *et al.* Spin Hamiltonians in magnets: Theories and computations. *Molecules* **26** (2021).

5 Buckingham, A. D. Molecular quadrupole moments. *Q. Rev. Chem. Soc.* **13**, 183-214 (1959).

6 Halasyamani, P. S. & Poeppelmeier, K. R. Noncentrosymmetric oxides. *Chem. Mater.* **10**, 2753-2769 (1998).

7 Miller, S. C. & Love, W. F. *Tables of irreducible representations of space groups and co-representations of magnetic space groups.* (Pruett Press, Boulder, 1967).

8 Campbell, B. J., Stokes, H. T., Tanner, D. E. & Hatch, D. M. Isodisplace: A web-based tool for exploring structural distortions. *J. Appl. Crystallogr.* **39**, 607-614 (2006).

9 Jana, M. K. *et al.* Organic-to-inorganic structural chirality transfer in a 2D hybrid perovskite and impact on Rashba-Dresselhaus spin-orbit coupling. *Nat. Commun.* **11**, 4699 (2020).
